# Supplementary material for: Intergenerational effects of early adversity on survival in wild baboons
Source: eLife. 2019 Sep 24;8:e47433. doi: 10.7554/eLife.47433 (PMC6759315; doi:10.7554/eLife.47433)
Supplement: Supplementary file 1. [file elife-47433-supp1.docx]

Table S1. Pearson correlation coefficients between binarized sources of early life adversity^a^

| r  (adj. p: Holm’s method) | Maternal Loss (Mom) | Maternal Loss (Offsp) | Low Maternal Rank (Mom) | Low Maternal Rank (Offsp) | Drought (Mom) | Drought (Offsp) | Large Group (Mom) | Large Group (Offsp) | Close-in-age Sibling (Mom) |
| --- | --- | --- | --- | --- | --- | --- | --- | --- | --- |
| Maternal Loss (Mom) | --- |  |  |  |  |  |  |  |  |
| Maternal Loss (Offsp) | 0.12^b^  0.07^d^ | --- |  |  |  |  |  |  |  |
| Low Maternal Rank (Mom) | -0.07  1.00 | 0.07  1.00 | --- |  |  |  |  |  |  |
| Low Maternal Rank (Offsp) | 0.07  1.00 | 0.06  1.00 | **0.40**  **<0.0001** | --- |  |  |  |  |  |
| Drought (Mom) | 0.06  1.00 | 0.06  1.00 | -0.02  1.00 | 0.04  1.00 | --- |  |  |  |  |
| Drought (Offsp) | 0.01  1.00 | 0.05  1.00 | -0.02  1.00 | 0.04  1.00 | -0.06  1.00 | --- |  |  |  |
| Large Group (Mom) | 0.13  **0.01** | 0.09  0.58 | -0.07  1.00 | 0.06  1.00 | -0.02  1.00 | -0.06  1.00 | --- |  |  |
| Large Group (Offsp) | -0.06  1.00 | 0.01  1.00 | -0.12  0.041 | -0.02  1.00 | -0.10  0.27 | 0.17  **0.0001** | 0.03  1.00 | --- |  |
| Close-in-Age Sibling (Mom) | -0.08  0.89 | 0.05  1.00 | 0.09  0.37 | -0.17  **0.0003** | -0.11  0.075 | 0.03  1.00 | -0.12  0.06 | 0.08  1.00 | --- |

^a^Correlations are calculated for binary measures of adversity within and between generations

^b^Pearson’s correlation coefficient (r), reported for each pair of variables. Coefficients > 0.2 appear in bold

^c^p-value for r, reported for each pair of variables

^d^p-value following Holm’s method for correcting for multiple testing

Table S2. Full mixed effects Cox proportional hazards model:

| **Generation** | **Parameter** | **Coefficient^a^** | **Hazard Ratio**  **(95% CI)** | **p value** | **Interpretation** |
| --- | --- | --- | --- | --- | --- |
| *Maternal* | Maternal Loss | 0.39 | 1.48  (1.12-1.95) | **0.006** | Offspring survived less well if their mother experienced maternal loss during her early life. |
|  | Close-In-Age Younger Sibling | 0.33 | 1.39  (1.03-1.89) | **0.03** | Offspring survived less well if their mother had a close-in-age younger sibling during her early life. |
|  | Low Maternal Rank | 0.22 | 1.25  (0.89-1.76) | 0.19 |  |
|  | Drought | 0.14 | 1.15  (0.77-1.71) | 0.50 |  |
|  | High Social Density | -0.12 | 0.89  (0.52-1.51) | 0.66 |  |
| *Offspring* | Maternal Loss | 0.67 | 1.95  (1.51-2.54) | **5x10^-7^** | Offspring survived less well if their mother died within four years of their birth. |
|  | Low Maternal Rank | 0.35 | 1.43  (1.05-1.94) | **0.03** | Offspring survived less if well if they were born to a low-ranking mother. |
|  | Drought | -0.29 | 0.75  (0.52-1.08) | 0.12 |  |
|  | High Social Density | -0.07 | 0.93  (0.71-1.22) | 0.61 |  |

^a^In all cases, positive coefficients indicate a higher hazard ratio in the presence of the adverse condition

Table S3. AIC values from models of offspring survival that included fixed effects of maternal age, offspring sex or interactions between age or sex and maternal adversity.

| **Model** | **AIC** |
| --- | --- |
| Reduced model (Table 2) | 3203.6 |
| Reduced model + offspring sex | 3205.5 |
| Reduced + offspring sex * mother exp. maternal loss | 3205.4 |
| Reduced + offspring sex * mother exp. close-in-age sib | 3207.4 |
| Reduced + both sex interactions | 3207.4 |

Table S4. Model output for a mixed effect cox proportional hazards model of offspring survival that additionally includes a fixed effects of the age of the mother at the time of the offspring’s birth.

| **Generation** | **Parameter^a^** | **Coefficient** | **Hazard Ratio**  **(95% CI)** | **p value** | **Interpretation** |
| --- | --- | --- | --- | --- | --- |
|  | Mother’s age at offspring birth | -0.002 | 1.00  (0.97-1.03) | **0.90** |  |
| *Maternal* | Maternal Loss | 0.35 | 1.43  (1.07-1.87) | **0.012** | Offspring survive less well if their mother experienced maternal loss during her early life. |
|  | Close-in-age Younger Sibling | 0.34 | 1.40  (1.05-1.89) | **0.024** | Offspring survive less well if their mother had a close-in-age younger sibling during her early life. |
| *Offspring* | Maternal Loss | 0.67 | 1.96  (1.49-2.57) | **8x10^-7^** | Offspring survive less well if they experienced maternal loss within four years of their birth. |
|  | Low Maternal Rank | 0.43 | 1.54  (1.17-2.03) | **0.002** | Offspring survive less well if they were born to a low-ranking mother. |

Table S5. Model output for a mixed effect cox proportional hazards model of offspring survival that additionally includes a fixed effects of the age of the mother at the time of the offspring’s birth and interactions between maternal age and sources of maternal early adversity.

| **Generation** | **Parameter^a^** | **Coefficient** | **Hazard Ratio**  **(95% CI)** | **p value** | **Interpretation** |
| --- | --- | --- | --- | --- | --- |
|  | Mother’s age at offspring birth | -0.001 | 1.00  (0.96-1.04) | **0.96** |  |
| *Maternal* | Maternal Loss | 0.34 | 1.40  (0.66-2.94) | **0.38** |  |
|  | Close-in-age Younger Sibling | 0.40 | 1.49  (0.63-3.14) | **0.33** |  |
|  | Mother’s age*Maternal Loss | 0.002 | 1.00  (0.94-1.07) | **0.96** |  |
|  | Mother’s age*Close-in-age Sibling | -0.006 | 1.00  (0.92-1.07) | **0.88** |  |
| *Offspring* | Maternal Loss | 0.67 | 1.96  (1.49-2.57) | **1x10^-6^** | Offspring survive less well if they experienced maternal loss within four years of their birth. |
|  | Low Maternal Rank | 0.43 | 1.54  (1.17-2.03) | **0.002** | Offspring survive less well if they were born to a low-ranking mother. |

Table S6. Alternative mixed effects survival model that includes cumulative maternal adversity instead of multivariate adversity conditions (R^2^=0.07, log likelihood = -1598)

| **Generation** | **Parameter** | **Coefficient** | **Hazard Ratio**  **(95% CI)** | **p value** | **Interpretation** |
| --- | --- | --- | --- | --- | --- |
| Maternal | Cumulative Maternal Adversity | 0.27 | 1.31  (1.12-1.52) | **0.0007** | Offspring survived less well if their mother experienced more adversity during her early life. |
| Offspring | Maternal Loss | 0.66 | 1.94  (1.50-2.52) | **6x10^-7^** | Offspring survived less well if their mother died within four years of their birth. |
|  | Low Maternal Rank | 0.30 | 1.36  (1.03-1.78) | **0.03** | Offspring survived less if well if they were born to a low ranking mother. |

Table S7. Final reduced models of offspring survival written in R syntax.

| **Adversity Model, results shown in Figure 1 and Table 2:** |
| --- |
| Surv(Age_4, Death_4)~ML+MS+OL+OR+(1\|Maternal ID)+(1\|Grandmaternal ID) |
|  |
| **Model of offspring survival as a function of maternal loss, results shown in Figure 2:** |
| Surv(Age_2, Death_2)~ML+(1\|Maternal ID) |
|  |

ML: Maternal loss in the mother’s generation

MS: Maternal exposure to a close-in-age younger sibling

OL: Maternal loss in the offspring’s generation

OR: Low maternal rank in the offspring’s generation

Age_4/Age_2: Offspring age at death/censor. Maximum age = 4 or 2 depending on analysis

Death_4/Death_2: Binary indicator of whether death occurred at the offspring’s listed age. (1= death, 0 = censored)
